# Microarray-based gene expression profiles of silkworm brains

**DOI:** 10.1186/1471-2202-12-8

**Published:** 2011-01-19

**Authors:** Ling Gan, Xilong Liu, Zhonghuai Xiang, Ningjia He

**Affiliations:** 1 The Key Sericultural Laboratory of Agricultural Ministry, College of Biotechnology, Southwest University, Beibei, Chongqing 400715, PR China

## Abstract

**Background:**

Molecular genetic studies of *Bombyx mori *have led to profound advances in our understanding of the regulation of development. *Bombyx mori *brain, as a main endocrine organ, plays important regulatory roles in various biological processes. Microarray technology will allow the genome-wide analysis of gene expression patterns in silkworm brains.

**Results:**

We reported microarray-based gene expression profiles in silkworm brains at four stages including V7, P1, P3 and P5. A total of 4,550 genes were transcribed in at least one selected stage. Of these, clustering algorithms separated the expressed genes into stably expressed genes and variably expressed genes. The results of the gene ontology (GO) and Kyoto encyclopedia of genes and genomes (KEGG) analysis of stably expressed genes showed that the ribosomal and oxidative phosphorylation pathways were principal pathways. Secondly, four clusters of genes with significantly different expression patterns were observed in the 1,175 variably expressed genes. Thirdly, thirty-two neuropeptide genes, six neuropeptide-like precursor genes, and 117 cuticular protein genes were expressed in selected developmental stages.

**Conclusion:**

Major characteristics of the transcriptional profiles in the brains of *Bombyx mori *at specific development stages were present in this study. Our data provided useful information for future research.

## Background

The silkworm, *Bombyx mori, *is a holometabolous insect that has four distinct life stages including embryo, larva, pupa, and moth. It is a model organism for Lepidoptera in molecular genetics and functional genomics and has greatly contributed to understanding of the mechanisms governing metamorphosis and diapause [1]. Lepidoptera represent a diverse group of agricultural insect pests of food and fiber crops worldwide. Hence, gaining a thorough understanding of gene function in Lepidoptera is a critical step when developing new and targeted methods of pest control. The insect brain is a 'supraesophageal ganglion' which is interconnected by paired circumesophageal connectives with subesophageal ganglion(SG)[2]. The insect brain is an important part of neurosecretory system. A large number of neuropeptide precursor genes have been characterized in insect brains [3]. As their major secreted molecules, neuropeptides play multiple functions. Brain neuropeptides are mainly used for cell-to-cell communication by multicellular organisms and play essential roles in regulating the growth and development of insects [3-7]. In 1947, Williams proved that the brain hormone (ie, PTTH) turns on the prothoracic glands which secrete a hormone directly responsible for stimulating the development of peripheral tissues [5]. A recent study showed that myosuppressin purified from pupal brains of *B. mori *functioned as a prothoracicostatic hormone and probably played an important role in controlling the development of silkworm [7]. Additional evidence indicated that neuropeptides including corazonin, FLRFamides, and myoinhibitory peptides (MIPs) were involved in the orchestration of the ecdysis behavioral sequence [8-10]. Research of targeted ablation of crustacean cardioactive peptide (CCAP) neuropeptide-containing neurons found that CCAP is also a key regulator of ecdysis and circadian regulation of *Drosophila *[11]. One study also revealed that *Bombyx *prothoracicostatic peptides whose gene transcripts were most prominent in brains of *Bombyx *activate the sex peptide receptor expressed in the prothoracic gland to regulate ecdysteroid biosynthesis [12]. Even single neuropeptide can be multifunctional and be expressed by different nerve cells at different stages. For instance, both immunohistochemistry and in situ hybridization techniques showed that different cell types expressed CCAP at specific developmental stages [13,14]. So studies related to the neuropeptide genes expression profiles in brains of silkworm will provide invaluable information. In our study, to provide new insights into activation of the ecdysis sequence during pupation and to better understand the regulating mechanisms during larval-to-pupal metamorphosis of silkworm, we investigated the gene expression profiles in the silkworm brain at selected stages V7, P1, P3, and P5 which are critical to such biological processes.

The technology of microarray allows the monitoring of expression of hundreds to thousands of genes simultaneously [15]. Importantly, this approach provides clues for elucidating the functions of genes underlying specific processes and identifying candidate genes predicted to regulate processes of interest. Several researchers have used microarray to study the gene expression patterns of selected tissues and developmental stages of silkworm. A 22,987 70-mer oligonucleotide microarray was designed and used to survey the gene expression in multiple silkworm tissues from mid-fifth instar larvae [16]. Huang *et al *reported a genome-wide analysis of silkworm host response to pathogen *Bacillus bombyseptieus *infection with microarrays [17]. Using an oligonucleotide microarray containing 23,134 probes, Liang *et al *studied the gene expression patterns in epidermal cells of silkworm [18]. An oligonucleotide microarray containing 34,631 oligonucleotide probes was used to analyze the global transcriptional profiles of host genes in *Bm*NPV-infected silkworm cells [19]. Recently a microarray system comprising 22,987 oligonucleotide 70-mer probes was employed to compare differentially expressed genes in the midguts of *Bm*CPV-infected and normal silkworm larvae [20]. However, the expression profiles of silkworm brains have not been reported yet.

This paper described the expression profiles of silkworm brains for the first time at V7, P1, P3 and P5 stages. A total of 4,550 genes were transcribed in at least one selected stage. The expression of several categories of genes encoding neuropeptide precursor, neuropeptide-like precursor (NPLP), and cuticular protein was observed. These results provided useful information and expanded our knowledge of the gene expression patterns of silkworm brains.

## Results

### Developmental expression profiles of genes in silkworm brains

Current oligonucleotide microarrays containing 23,224 probes were used to investigate the developmental expression profiles of genes in brains at V7, P1, P3, and P5. The complete sets of raw and normalized data from this study have been deposited in the Gene Expression Omnibus (GEO) repository (accession number GSE25342). Based on the criterion of signal intensity more than 800, 3,436, 3,048, 3,468, and 3,409 of genes were transcribed at V7, P1, P3, and P5, respectively. A total of 4,550 (19.60%) genes were expressed in at least one selected stage.

**Figure 1:**
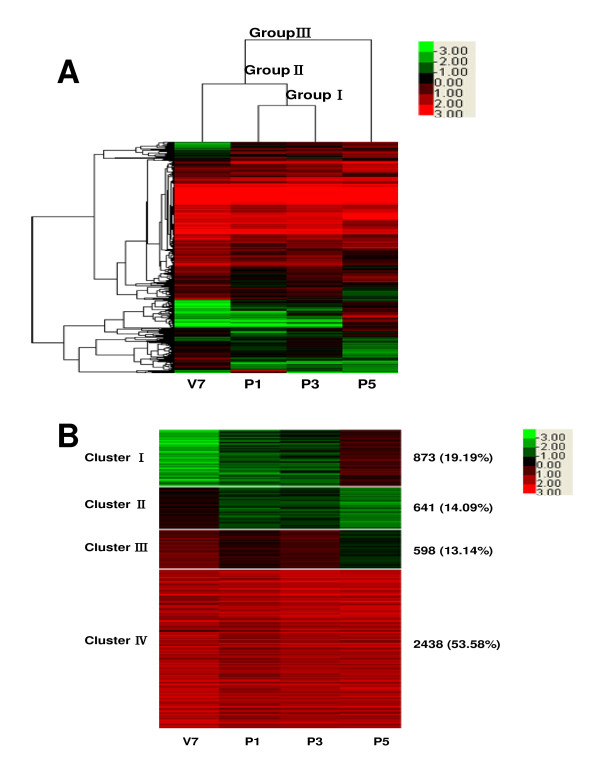
**Heat map of clustering of 4,550 genes expressed in silkworm brain tissues**. Genes expressed with signal intensity more than 800 were included in this analysis. Clustering was done using Cluster 3.0 software. The colors in the map display the relative values of all tiles within the four selected developmental stages; Green indicates the lowest expression, black indicates the intermediate expression, and red indicates the highest expression. The numerical values give the actual values on a log 2 scale, which are associated with each color. The color scale bar is shown at the top right corner of the figure. **A**: Genes clustered by the given 4 developmental stages (clustering type: hierarchical clustering, Distance metric: Pearson correlation). **B**: Genes grouped into four clusters on the basis of the similarity of expression (clustering type: K-means clustering, Distance metric: Pearson correlation). The number of the expressed genes and the percentage in each cluster are indicated.

Clustering algorithms and Treeview were used to analyze the expression profiles of these 4,550 genes. From the heat map of hierarchical clustering, as shown in Figure 1A, we found that two (P1 and P3) of the selected 4 stages were clustered together, indicating that genes shared similar expression profiles in silkworm brains in these stages. K-means clustering was further performed (Figure 1B), resulting in 4,550 genes divided into four clusters. Clusters I, II, III, and IV had 873 (19.2%), 641 (14.1%), 598 (13.1%), and 2,438 genes (53.6%), respectively. Genes in the four clusters showed distinctly different expression patterns. In cluster I, high levels of expression were observed in P5. In clusters II and III, it was found that genes were more abundantly expressed in V7 and P3 than those in P1 and P5. It is worthwhile to mention that genes in cluster IV were highly expressed in four stages. Therefore, genes in cluster IV were considered as the stably expressed genes that is convenient for description.

### Stably expressed genes in silkworm brains along with development

**Figure 2:**
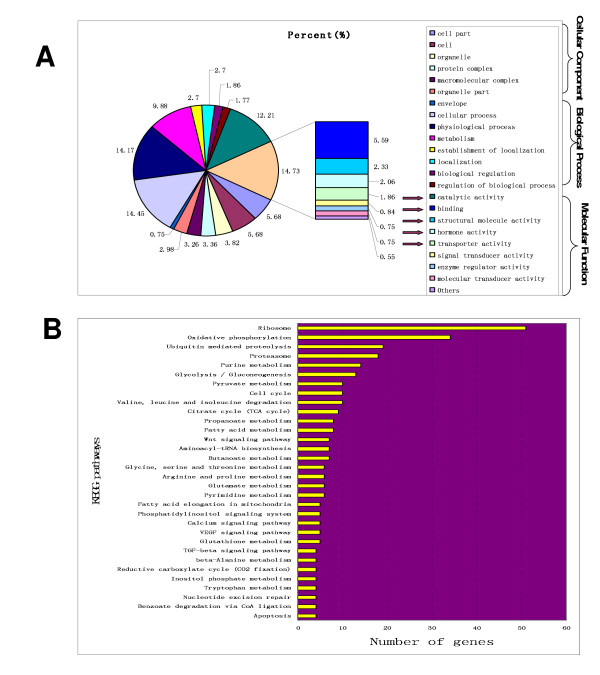
**Gene ontology categories and KEGG pathways of stably expressed genes by automatic GO analysis of Molecule Annotation System (MAS2.0)**. **A**: Gene ontology categories for stably expressed genes. Genes were classified into cellular component, molecular function, and biological process. Prunosus arrow heads indicate the predominant molecular functions among the stably expressed genes. **B**: KEGG pathways for stably expressed genes. Those pathways which involve more than four genes were selected for mapping and were listed according to the number of genes.

Genes whose expression was detected in silkworm brain were separated into two parts, stably expressed genes and variably expressed genes. Automatic GO analysis of Molecule Annotation System (MAS2.0) was used to analyze the stably expressed genes. As shown in Figure 2A, they were divided into three categories including molecular function, biological process, and cellular component. The molecular function category mainly consisted of five subcategories including catalytic, binding, structural molecule, hormone, and transporter activity. In the catalytic subcategory, a large number of genes encoding enzymes were expressed including helicase, hydrogen-transporting ATP synthase, oxidoreductase, protein kinase, hydrolase, ATPase, threonine- and serine-type endopeptidase, transferase, and ubiquitin-protein ligase. Twenty-two neuropeptide genes were found in the subcategory of hormone activity. Of these, eight bombyxin genes were detected. Cuticular protein genes and ribosomal genes were predominant in the structural molecule subcategory. It should be noted that prior to this study the expression of 25 cuticular protein genes had not been detected.

Using automatic analysis of KEGG pathways in MAS2.0, we found 2,438 stably expressed genes involved in 90 pathways including nine signaling pathways such as Wnt, Phosphatidylinositol, Calcium, vascular endothelial growth factor (VEGF), insulin, and transforming growth factor beta (TGF-beta ) signaling pathways. Figure 2B also showed that a large number of expressed genes were involved in ribosomal and oxidative phosphorylation pathways. The automatic GO and KEGG analysis of MAS2.0 for stably expressed genes was provided in Additional file 1 and 2, respectively.

### Variably expressed genes in silkworm brains along with development

**Figure 3:**
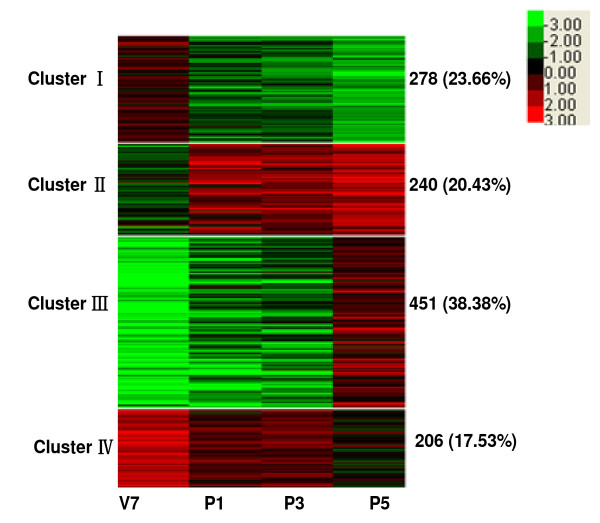
**Heat map of K-means clustering of 1175 variably expressed genes in four developmental stages**. Clustering was done using Cluster 3 software (clustering type: K-means clustering, Distance metric: Pearson correlation). The colors in the map display the relative values of all tiles within four selected developmental stages; Green indicates the lowest expression, black indicates intermediate expression, and red indicates the highest expression. The numerical values give the actual values on a log 2 scale, which are associated with each color. The color scale bar is shown at the top right corner of the figure. Genes were grouped into four clusters on the basis of the expression similarity. The number of the expressed genes and the percentage in each cluster are indicated.

Of the 4,550 genes expressed in brains at four stages, 1,175 variably expressed genes were screened at a four-fold cut-off value. The ratio of more than 4 or less than 0.25 represents up- and down- regulated expression, respectively. As shown in Figure 3, K-means clustering divided the 1,175 genes into four clusters. Genes in cluster I were highly expressed at V7, whereas genes in cluster III were highly expressed at P5. Genes in cluster II were highly expressed in a pupal-biased manner but the expressions of genes in cluster IV were relatively repressed in pupal stages. In cluster I, eleven neuropeptide genes, one NPLP gene, and one neurogenin related protein gene were found. Genes encoding antennal binding protein, chemosensory related protein, and five cytochrome P450s were also detected. Interestingly, some immune-related genes were detected including two immulectin genes and one immune-related Hdd11 gene. In addition, the expressions of MBF2 gene, aquaporin gene, cuticular protein genes, TIME-EA4 gene, yellow protein gene, and wing disc-specific protein genes were observed in this cluster. In cluster II, nine cuticular protein genes as well as the genes encoding carboxylesterase, chitin binding protein, lipase, silkworm trachealess, insulin-related peptide binding protein were identified. In cluster III, the expressions of a pheromone biosynthesis activating neuropeptide (PBAN) gene, 45 cuticular protein genes, and a ATP-dependent RNA helicase DDX18 (DEAD box protein 18) gene were detected. In cluster IV, a neuropeptide AKH2 gene, a NPLP gene, and two cuticular protein genes were transcribed. The homology Blast results for 1,175 variably expressed genes were provided in Additional file 3.

### The expression profiles of neuropeptide genes in silkworm brains

**Figure 4:**
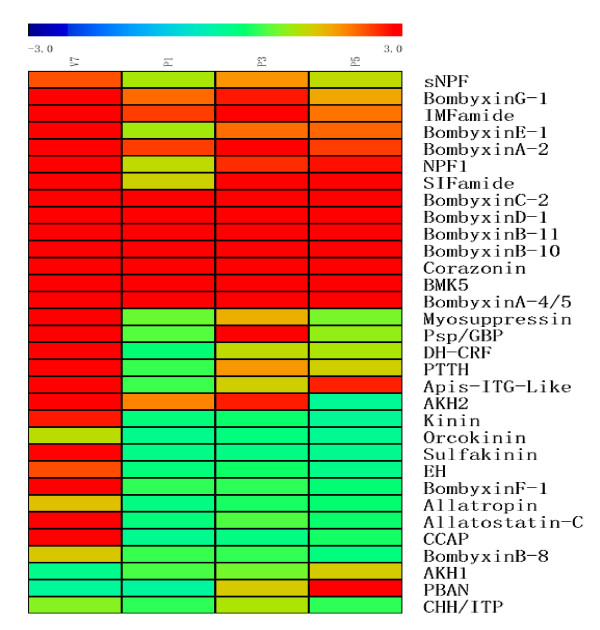
**Heat map of Stringent QT (quality threshold) clustering of expressed neuropeptide hormone genes**. Clustering was done using Mev software and was displayed by rainbow scheme. The colors in the map display the relative values of all tiles within four selected developmental stages; Blue indicates the lowest expression and red indicates the highest expression. The numerical values give the actual values on a log 2 scale, which are associated with each color. The color scale bar is shown at the top corner of the figure. The genes names are indicated.

**Figure 5:**
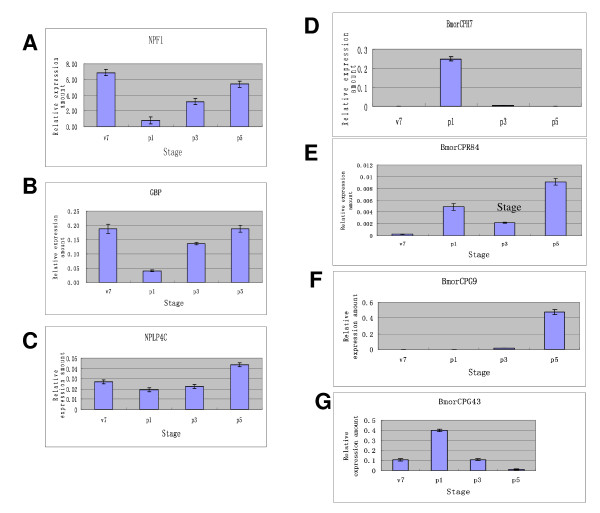
**qRT-PCR results of seven genes**. **A**: NPF1; **B**: GBP; **C**: NPLP4E; **D**: BmorCPH7; **E**: BmorCPR84; **F**: BmorCPG9; **G**: BmorCPG43.

Based on the sequence homology and the structure conservation in the neuropeptides from other invertebrates, 43 putative neuropeptide genes rather than bombyxin genes were annotated by searching the silkworm genome and national center for biotechnology information (NCBI) databases. The corresponding gene numbers were listed in Additional file 4. Fifty-two oligonucleotide probes were designed for all silkworm putative neuropeptide genes including 11 bombyxin genes. Three pairs of genes, namely, NPF1a and NPF1b genes, CAPAa and CAPAb genes, and bombyxinA-4 and A-5 genes shared the same probes because of the high sequence similarity. Stringent quality threshold (QT) clustering algorithm analysis was carried out to display the developmental expression profile of neuropeptide genes. As shown in Figure 4, the expression of 32 neuropeptide genes was detected. Among them, 14 neuropeptide genes were expressed at all stages including short neuropeptide F (sNPF), IMFamide, neuropeptide F1 (NPF1), SIFamide, corazonin, BMK5, and eight bombyxin genes with conservative motif of A, B, C, D, E, G subfamilies. Genes for kinin, orcokinin, sulfakinin, eclosion hormone (EH), allatropin, allatostatin-C, CCAP, bombyxinB-8, and bombyxinF-1 were expressed only at V7. The pheromone biosynthesis activating neuropeptide (PBAN) gene was expressed at a relatively higher level at P5. The expression patterns of two genes, NPF1 and growth-blocking peptide (GBP), were further confirmed by real-time PCR. As shown in Figure 5A and 5B, the results of real-time PCR showed that their expression profiles were consistent with the results of the microarray.

### The expression profiles of neuropeptide-like precursor genes in silkworm brains

**Figure 6:**
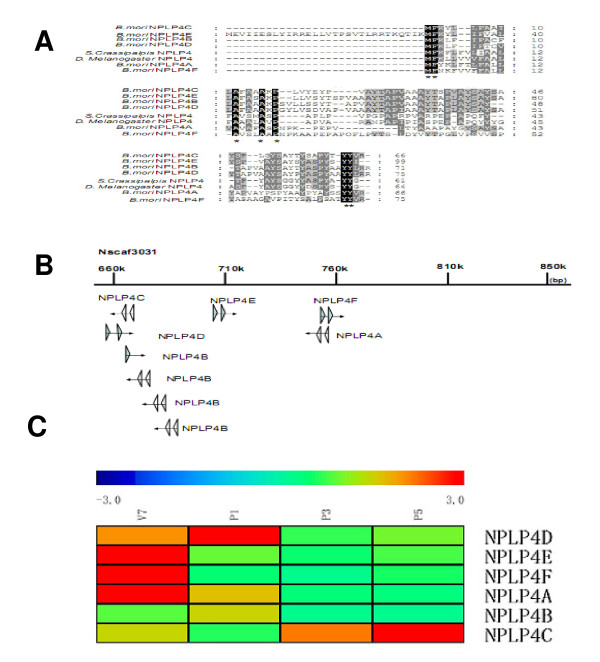
**Heat map of Stringent QT (quality threshold) clustering, the sequence BLAST results and the organization of the NPLP genes**. **A**: Amino acid sequence BLAST analysis of *Bombyx mori *theoretical NPLPs (accession number: HQ386680-HQ386685) with those sequences from *Sarcophaga Crassipalpis *NPLP4 (accession number: EU526381.1), *Drosophila Melanogaster *NPLP4 (accession number: NM_144095.1). Amino acids conserved in NPLP are highlighted in dark and light gray. *. **B**: Genomic organization of NPLP genes. Triangles correspond to the exons. Arrows indicate transcriptional direction. Nscaf: Scaffold data assembled by Japanese and Chinese groups. **C**: Heat map of Stringent QT (quality threshold) clustering of six expressed NPLP genes. Clustering was done using Mev software and was displayed by rainbow scheme. The colors in the map display the relative values of all tiles within four selected developmental stages; Blue indicates the lowest expression and red indicates the highest expression. The numerical values give the actual values on a log 2 scale, which are associated with each color. The color scale bar is shown at the top corner of the figure. The names of genes are indicated.

Neuropeptide like precursor (NPLP) genes had been discovered in the central nervous system of *Drosophila melanogaster *and *Sarcophaga crassipalpis *[21,22]. Six putative NPLP4 genes were found in silkworm genome database by BLAST searches (Figure 6A). Of these, NPLP4B has four copies. All the NPLP4 proteins have three highly conserved amino acids YYX (x designated to L, V, I, G) at the C-terminal. The silkworm NPLP4 genes are distributed in chromosomes 11 in a narrow region at the nscaf 3031, as shown in Figure 6B.

Six oligonucleotide probes were designed for these NPLP4 genes. The result of QT clustering algorithm analysis showed that they had various expression patterns in silkworm brains (Figure 6C). For example, the expression of NPLP4C gene was repressed at P1, whereas it expressed at a higher level at P5. The real-time PCR data for this gene (Figure 5C) showed a similar expression pattern.

### The expression profiles of cuticular protein genes in silkworm brains

**Figure 7:**
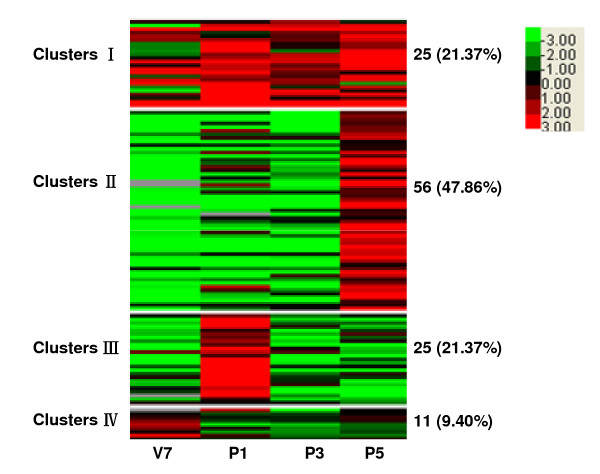
**Heat map of hierarchical clustering of 117 cuticular protein genes in four developmental stages**. Clustering was done using Cluster 3 software. The colors in the map display the relative values of all tiles within four selected developmental stages; Green indicates the lowest expression, black indicates the intermediate expression, and red indicates the highest expression. The numerical values give the actual values on a log 2 scale, which are associated with each color. The color scale bar is shown at the top right corner of the figure. Genes grouped into four clusters on the basis of the similarity of expression (clustering type: K-means clustering, Distance metric: Pearson correlation). The number of the expressed genes and the percentage in each cluster are indicated.

In the present microarray data, prominent among the brain-expressed genes were cuticular protein genes. The expressions of 117 cuticular protein genes were detected in silkworm brains. K-means clustering was performed (Figure 7), resulting in 117 cuticular protein genes divided into four clusters. Cuticular protein genes in cluster I were highly expressed from P1 to P5. The expressions of genes in cluster II, III, and IV were almost restricted to P5, P1 and V7, respectively. The results were also supported by the real-time PCR data (Figure 5D, 5E, 5F, 5G).

In silkworm, cuticular protein genes are divided into six families including RR (RR-1, RR-2, RR-3), Tweedle, CPF, CPFL, CPG, and CPH families based on conserved motifs [18]. To better understand the expression profiles of cuticular protein genes bearing particular motifs in the silkworm brains, the number of genes in each cluster and the distribution in different families were summarized (Table 1). The cuticular protein genes belonging to RR, Tweedle, CPFL, CPG, and CPH families were exclusively expressed. But the expression of CPF gene was undetectable.

**Table 1:** Expression profiles of cuticular protein genes bearing particular motifs

	RR Consensus			Tweedle	CPF	CPFL	CPG	CPH	Total
							
	RR-1	RR-2	RR-3						
Cluster I	4	5	0	0	0	1	9	6	25
Cluster II	10	21	0	2	0	1	14	8	56
Cluster III	1	9	1	1	0	1	0	12	25
Cluster IV	4	0	0	0	0	0	2	5	11

Total	19	35	1	3	0	3	25	31	117

## Discussion

In the present study, we designed 90 additional oligonucleotide probes for previously unpredicted genes and monitored gene expression profiles of silkworm brains isolated from four developmental stages using the oligonucleotide microarray containing 23,224 probes. The expression of 4,550 genes was detected in these stages. It resulted in the first microarray-based gene expression profiles of a lepidopteran insect brain.

Genes whose expressions were detected in silkworm brains were separated into two parts, stably expressed genes and variably expressed genes. Of previously reported 306 *B. mori *house-keeping genes [16], 304 genes were transcribed in the category of our stably expressed genes. The remaining two house-keeping genes, mitochondrial citrate synthase precursor gene and neurofilament gene were classified to cluster III and cluster I, respectively (Figure 1B). The results of the GO and KEGG analysis of stably expressed genes showed that the ribosomal and oxidative phosphorylation pathways were principal pathways, suggesting that metabolisms of protein were probably prominent in silkworm brains.

K-means clustering was carried out to study the expression patterns of 1,175 variably expressed genes. The expressions of a neurogenin related protein gene, an antennal binding protein gene, and a chemosensory protein related gene were detected in cluster I. A mouse neurogenin related protein was once reported to function as a vertebrate neuronal determination factor analogous to those of the *Drosophila *proneural genes [23]. Antennal binding protein and chemosensory protein are responsible for sensory signaling and might be required for the correct recognition of some odors [24-26]. Our analysis detected the expression of five cytochrome P450 genes in cluster I, none of which are silkworm halloween genes, which are involved in biosynthesis of 20-hydroxyecdysone (20E) from cholesterol [27-32]. Since the cytochrome P450 gene superfamily consists of 86 putative members [33], the expression of these five cytochrome P450 genes in silkworm brain might provide clues for their functional study. The genes in cluster III were highly expressed at P5 stage including a PBAN gene and a DDX18 gene. PBAN is secreted from the subesophageal ganglion and is required for pheromone synthesis [34]. Along with PBAN peptide, a diapause hormone (DH) and three additional FXPR/KL neuropeptides (NPs: a, b, g) were post-translationally processed from a polyprotein precursor well conserved in moths [35,36]. DDX18 proteins are putative RNA helicases that function in all aspects of RNA metabolism, including translation, ribosome biogenesis, and pre-mRNA splicing [37]. In cluster IV, the reduced gene expression in pupal stage may be responsible for the decreased titer of molting hormone [38,39].

The expression profiles for neuropeptide genes in silkworm brains revealed that there was no correlation between neuropeptide expression profile and conservative motif. For instance, eight bombyxin genes were expressed at all stages, and two bombyxin genes were only expressed at V7, a time for initiation of pupation. Bombyxin was purified from the heads of the male adult *Bombyx mori *as a factor that stimulated the prothoracic glands (PGs) of *Samia cynthia ricini*, but failed to stimulate the PGs of *Bombyx *itself to synthesize and release ecdysteroid [40-42]. Moreover, bombyxin neuropeptides were also involved in various biological processes in silkworm such as metabolism and growth [43,44]. Another neuropeptide gene with highest expression level at V7 is PTTH gene. PTTH is necessary for the induction of larval-pupal metamorphosis and stimulates the PGs to synthesize and release ecdysteroids [4,5]. In our study the expression peak of PTTH gene on V7 probably shows its ability to stimulate ecdysone biosynthesis, but whether it is same for two bombyxin genes will be further explored. Besides two bombyxin genes and a PTTH gene, many neuropeptide genes were highly expressed at V7 including the genes encoding eclosion hormone (EH), CCAP, kinin, orcokinin, sulfakinin, allatropin, and allatostatin-C, suggesting that in the face of impending pupation, these neuropeptide genes were abundantly expressed. Previous studies have showed that EH, CCAP, and ecdysis triggering hormone (ETH) probably coordinated the sequence of pre-ecdysis, ecdysis, and postecdysis behaviors [45-47]. Corazonin, FLRFamides, and MIPs were also involved in orchestration of ecdysis sequence [8-10]. Our data will facilitate further exploration of the functions and the interaction of neuropeptides in ecdysis sequence. Furthermore, our data clearly showed that some neuropeptide genes with opposing effects were expressed in a similar pattern in the silkworm brains, for example, PTTH versus myosuppressin and allatropin versus allatostatin-C. In the PGs, PTTH stimulates ecdysteroidogenesis whereas bommo-myosuppressin (BMS) inhibits the same process [5,7]. But BMS was not the first reported inhibitor to PTTH-stimulated ecdysteroidogenesis, a prothoracicostatic peptide (PTSP) characterized by Hua *et al*. was proved to have such a function at both the spinning and feeding stages in the brain of silkworm [48]. BMS and PTSP regulated the PGs activity via different receptors, and BMS had a higher prothoracicostatic activity on larval PGs than PTSP [7]. According to our data, the expression of BMS at V7 is more abundant than that of PTSP.

NPLP genes were first identified by analysis of the peptidome of the *D. melanogaster *larval central nervous system [21]. However until now, none of NPLPs had a function assigned to them except that the expression of NPLP1 in the brain and ventral nerve cord of *D. melanogaster *was observed [49]. In addition, *S. crassipalpis *NPLP4 was uniquely expressed during diapause which suggested its potential role in diapause initiation and maintenance [22]. In the silkworm genome database, the putative NPLP1 and NPLP4 genes were found by homology search. According to the present microarray data, the expressions of six NPLP4 genes were detected in silkworm brains. EST evidence has shown that NPLP4E was present in epidermis, ovary and prothoracic gland of silkworm (EST: BP123504; BB984435; BY936052). Liang *et al. *also reported that several NPLPs, which correspond to the NPLP4B, NPLP4E, and NPLP4F in our study, were highly expressed in epidermal tissues of silkworm when molting was initiated [18]. All these results indicated that the expression of NPLP4 genes was not tissue-specific. The diverse expression patterns of these NPLP4 genes in the different tissues of silkworm implied they may function in various physiological processes.

Cuticular proteins are usually considered as major components of the insect cuticle. But in our study, a large number of cuticular protein genes were expressed in brains of silkworm at P1 and P5. The observed expression of cuticular protein genes in brains was not a surprise since their EST data had been identified in multiple internal organs such as ovary, brain, and posterior silk gland [50].

Our data revealed massive expression of a majority of cuticular protein genes at pupal stages, especially at P1 and P5. A reasonable explanation for this observation is that these cuticular protein genes were transcribed and immediately translated to proteins participating in the morphologic and synaptic reorganization of brains acquiring new features as required by development [51]. The morphologic images of brains at four stages were provided in Additional file 5. The silkworm brain is composed of an optical lobe (OL), antennal lobe (AL), and central brain (CB) [52-54]. During metamorphosis, pronounced developmental alterations of the morphology of all these were observed in *Manduca sexta *[55]. Our data indicated that the reorganization of silkworm brain is very active at pupal stages. In addition, our data clearly showed no correlation between the expression profiles of cuticular protein genes and the presence of conserved motifs. This result is consistent with what was found in the expression profiles of cuticular proteins in silkworm epidermal tissues [18]. This led us to suspect that different cuticular protein genes were recruited to build the complex structures of silkworm brains.

## Conclusion

For the first time, we have surveyed gene expression profiles in silkworm brains at V7, P1, P3 and P5 by means of a whole-genome oligonucleotide microarray. Based on our microarray data, a total of 4,550 genes were activated, among those, 53.58% genes didn't change expression acutely during tested stages, which mainly were involved in protein metabolism. Manual screening of 1,175 variably expressed genes revealed different expression profiles reflecting intense stage-specific characteristics. Thirty-two neuropeptide genes, six neuropeptide-like precursor genes and 117 cuticular protein genes were expressed in selected developmental stages, displaying diverse expression patterns. This dataset will provide some important clues and novel insights for further research.

## Methods

### Silkworm rearing, tissue isolation and RNA extraction

Silkworm larvae (P50 strain) were reared on fresh mulberry leaves, 12 h light/12 h dark photoperiod at 24°C~26°C, and with 70-85% relative humidity in the Institute of Sericulture and System Biology (Southwest University, China). Silkworm strain p50 grows through five instars until cocoon spinning which begins at the end of the fifth instar larva day 7. After spinning for three days, silkworms develop into the pupal stage. The synchronized animals were collected after each molting. Silkworm brains were excised from day-7 5^th ^instar larvae and day-1, 3, 5 pupae to extract the total RNAs. The isolation was carried out on ice and the purity of tissue preparations were checked with microscopy. Total RNA was isolated from the brains using Trizol reagent (Invitrogen, Carlsbad, CA, USA) according to the manufacturer's instructions. The concentration of RNA was spectrophotometrically determined.

### Oligonucleotide microarray design and construction

An updated silkworm oligonucleotide microarray was constructed by adding 90 new probes based on a previously designed microarray [18]. All these probes were designed by CapitalBio Corporation (Beijing, China) and were synthesized by MWG Biotech (Ebersberg, Germany). The microarray slide consisted of 48 blocks, each with 22 rows and 23 columns. Five housekeeping genes and eight yeast intergenic sequences were dotted in one block as positive and external controls, respectively. Dual channel micaroarray hybridization was performed with a Cy3-labeled control sample and Cy5-labeled test sample. Total RNA extracted from the whole body of silkworm at day 6 of the fifth instar larvae was served as a normalization control for data analysis.

### Amplification, labeling and array hybridization

The total RNAs were further purified using a NucleoSpin RNA clean-up kit (Macherey-Nagel, Germany). The amplification and labeling of mRNA were performed according to the previous studies [16,18]. Briefly, five micrograms of each RNA samples were first primed with 1 μl of 100 μM primer which contained T7 RNA polymerase promoter sequence at 70°C for 10 min, followed by reverse transcription at 42°C for 2 h in the presence of 200 U CbcScript (CapitalBio Corp, China). The second strand of cDNA was synthesized at 16°C for 2 h with the help of RNaseH and DNA polymerase. cRNA was synthesized by T7 Enzyme Mix (CapitalBio Corp, China) using the cDNA template. 2 μg of cRNA were primed with random primer at 65°C for 10 min, and then reversely transcribed at 25°C for 10 min and 37°C for 1.5 h in the presence of CbcScript II (CapitCapitalBio Corp, China). A CapitalBio cRNA Amplification and Labeling Kit (CapitalBio, Beijing, China) was used to label the Cy3- and Cy5-dCTP double-stranded cDNA. Cy5-dCTP or Cy3-dCTP were added at a final concentration of 120 μM of each dATP, dGTP, and dTTP and 60 μM dCTP and 40 μM Cy5-dCTP for test samples. For reference samples, Cy3-dCTP was employed. The Cy3- and Cy5-dCTP double-stranded cDNA were dissolved in 80 μl hybridization solution which contained 3 × SSC, 0.2%SDS, 5 × Denhart's, and 25% formamide. The hybridization was conducted in a closed chamber at 42°C over-night after the slides were covered with a LifterSlip™ coverslip (Erie Company, Portsmouth, NH, USA). After that, slides were washed three times using 0.2% SDS, 2x SSC at 42°C for 5 minutes and three times with 0.2 × SSC at room temperature for 5 minutes before signal scanning.

### Microarray data processing and analysis

The slides were scanned with a confocal LuxScan scanner (CapitalBio Corp) and the raw data were extracted using LuxScan™ 3.0 software (CapitalBio Corp). For dual-channels microarray data, the scanning setting for Cy3 and Cy5 channels were balanced by visual inspection of the external control spots. The LOWESS (Locally Weighted Scatterplot Smoothing) method was used to normalize the dual channel data using all the signals from the Cy5-labeled sample. The one with a fluorescent intensity higher than 800 after subtracting the background was considered as an expressed gene since the signal greater than that detection level was reliable. The expression of each gene was defined by the ratio of the original signal intensity divided by 800. The X-fold values were used in the following clustering analysis to display the expression of detected genes at various developmental stages. HCL (Hierarchical Clustering) and K-mean clustering analysis were carried out employing Cluster 3.0 software [56]. Mev software (version 4.2.01) was used for QT (quality threshold) clustering analysis [57]. The parameter setting for clustering analysis was based on the distance metric of the Pearson correlation and the average linkage method. TreeView software was used to display heat map of clustering results. KEGG pathway and GO category were automatically annotated by MAS2.0 analytical system maintained in CapitalBio Corp (http://bioinfo.capitalbio.com/mas/login.do). The sequences BLAST analysis was carried out using clustalx1.81 software.

### Quantitative real-Time PCR

To confirm the microarray data quantitative real-time PCR (qRT-PCR) was performed. Total RNAs were transcribed by M-MLV reverse transcriptase (Promega). The gene-specific primers were designed with Primer5.0 software. Each pair of primers was used to perform qRT-PCR after its efficacy (90-110%), correlation coefficient (≥95%) and specificity had been checked. qRT-PCR was performed in triplicate for each gene of interest in 20 μL reactions with reverse transcription product, 2 × PCR buffer, 50 × ROX Reference Dye, 100 nM of each of the forward and reverse primer, and RNase Free ddH2O. The PCR reaction was run on a ABI Prism 7000 Sequence Detection System (Applied Biosystems) using the following program: initial denaturation at 95°C for 10 sec, and 40 cycles of 95°C for 5 sec, 60°C for 31 sec. mRNA levels were quantified in relation to the expression of β-actin3.

## Abbreviations

V7: day 7 of the fifth instar larva; P1: day 1 of the pupa; P3: day 3 of the pupa; P5: day 5 of the pupa; SG: subesophageal ganglion; GO: gene ontology; KEGG: kyoto encyclopedia of genes and genomes; PTTH: prothoracicotropic hormone; MIPs: myoinhibitory peptides; CCAP: crustacean cardioactive peptide; MAS: molecule annotation system; NCBI: National Center for Biotechnology Information; QT clustering: quality threshold clustering; PBAN: pheromone biosynthesis activating neuropeptide; VEGF: vascular endothelial growth factor; TGF: transforming growth factor; NPLP: neuropeptide-like precursor; DDX18: DEAD box protein 18; 20E: 20-hydroxyecdysone; AKH: adipokinetic hormone; sNPF: short neuropeptide F; NPF1: neuropeptide F1; EH: eclosion hormone; GBP: growth-blocking peptide; PGs: prothoracic glands; BMS: bommo-myosuppressin; PTSP: prothoracicostatic peptide; ETH: ecdysis triggering hormone; DH (CRF-like): Corticotropin-releasing factor-like diuretic hormone; CHH: Crustacean hyperglycemic hormone; OL: optical lobe; AL: antennal lobe; CB: central brain; qRT-PCR: quantitative real-time polymerase chain reaction.

## Competing interests

The authors declare that they have no competing interests.

## Authors' contributions

NH conceived of the study and developed the study design. LG and XL carried out the analysis and LG drafted the manuscript. ZX participated in the study design and drafting of the manuscript. NH contributed to the critical revision of the manuscript. All authors read and approved the final manuscript.

## Supplementary Material

Additional file 1 **The automatic GO analysis of Molecule Annotation System for stably expressed genes**. This file contains three subsection including molecular function, biological process and cellular component of stably expressed genes in our microarray. It has multiple columns-Go term, total, Pvalue, Qvalue, protein and input symbol in each subsection. Some probes representing new genes have no Go term. Click here for file

Additional file 2 **The automatic KEGG analysis of Molecule Annotation System for stably expressed genes**. This file has multiple columns- pathway name, total, Pvalue, Qvalue, gene and symbol. Some probes representing new genes have no pathway name. Click here for file

Additional file 3 **The homology BLAST results for 1,175 viably expressed genes**. This file contains the homology BLAST results for 1,175 viably expressed genes in our microarray. It has multiple columns-input symbol, expression pattern, e-value and definition. Click here for file

Additional file 4 **The neuropeptide genes of silkworm**. This file has a table which contains all the detected silkworm neuropeptide genes in our microarray. The neuropeptide gene family, number for gene and accession numbers are also given. Click here for file

Additional file 5 **The morphologic images of silkworm brains at V7, P1, P3 and P5 stages**. The file contains the morphologic images of silkworm brains at V7, P1, P3 and P5 stages. The arrow heads indicate the optical lobe (OL), antennal lobe (AL), and central brain (CB), respectively. Click here for file
