# Supplementary material for: Microarray-based gene expression profiles of silkworm brains
Source: BMC Neurosci. 2011 Jan 19;12:8. doi: 10.1186/1471-2202-12-8 (PMC3032748; doi:10.1186/1471-2202-12-8)
Supplement: Additional file 4 — The neuropeptide genes of silkworm. This file has a table which contains all the detected silkworm neuropeptide genes in our microarray. The neuropeptide gene family, number for gene and accession numbers are also given. [file 1471-2202-12-8-S4.DOC]

**Neuropeptide genes in *Bombyx mori***

| **Neuropeptide gene family** | **Number for gene in Database** | **Accession numbers** |
| --- | --- | --- |
| AKH1 | BGIBMGA008722 | AB298930 |
| AKH2 | BGIBMGA008672 | AB298931 |
| AKH3 | gi:195182592 | AB298938 |
| Apis-ITG-like | gi:193248555 | AB298925 |
| AST- A | BGIBMGA014377 | AF309090 |
| AST- B | BGIBMGA009464 | AB073553 |
| AST- C | BGIBMGA005369 | AB362225 |
| AT | BGIBMGA011850 | AY970687 |
| BMK5 | BGIBMGA008978 | AB162718 |
| Bursicon α | BGIBMGA011022 | BN000691 |
| Bursicon β | BGIBMGA011086 | AY823260 |
| CAPAa | gi:191175143 | AB362227 |
| CAPAb | gi:191175145 | AB362228 |
| CCAP | BGIBMGA009606 | AB298937 |
| CCHamide | gi:194072591 | AB365354 |
| CHH | BGIBMGA004168 | AB031074 |
| Corazonin | BGIBMGA002280 | AB106876 |
| DH (Cal –like) | BGIBMGA011588 | AB362226 |
| DH (CRF-like) | gi:193248573 | AB298934 |
|  | gi:193248575 | AB298935 |
|  | gi:193248577 | AB298936 |
| EH | BGIBMGA006291 | D10135 |
| ETH | BGIBMGA009728 | NM_001172272.1 |
| FMRFamide | BGIBMGA009271 | AB234100.1 |
| Myosuppressin | BGIBMGA010073 | AU005976 |
| NPF2 | BGIBMGA009803 | AB298926 |
| NPF1 | gi:191175137 | AB362224 |
|  | gi:40861544 | CK500802 |
| IMFamide | gi:193248554 | AB298924 |
| Kinin | BGIBMGA010075 | AB298928 |
| Neuroparsin | BGIBMGA006409 | AB298927 |
| Orcoknin | BGIBMGA005422 | AB298932 |
| PBAN | BGIBMGA001651 | D16230 |
| PDH | gI:193248572 | AB298933 |
| Proctolin | BGIBMGA008345 | HQ386686 |
| PTTH | BGIBMGA000357 | D90082 |
| SIFamide | gi:193248552 | AB298923 |
| Tachykinin | BGIBMGA010076 | AB298929 |
| BombyxinG-1 | BGIBMGA011972 | NM_001128162.1 |
| BombyxinE-1 | BGIBMGA012294 | NM_001126261.1 |
| BombyxinA-2 | BGIBMGA012476 | HQ386687 |
| BombyxinC-2 | gi:1872817901 | D00792.1 |
| BombyxinD-1 | BGIBMGA011926 | AB003905 |
| BombyxinB-11 | gi:189458863 | NM_001128134.1 |
| BombyxinB-10 | gi:190341033 | NM_001128316.1 |
| BombyxinA-4 | gi:189458865 | NM_001128135.1 |
| BombyxinA-5 | gi:189458867 | NM_001128136.1 |
| BombyxinF-1 | BGIBMGA011930 | AB001049.1 |
| BombyxinB-8 | BGIBMGA011971 | NM_001128318 |
| sNPF | BGIBMGA012522 | AB330419 |
| Sulfakinin | BGIBMGA013558 | AB362223 |
| GBP | BGIBMGA014551 | AB064522.1 |
| GPA2 | gi:194072586 | AB365128 |
| GPB5 | BGIBMGA010342 | AB365129 |

AKH: Adipokinetic hormone, AST: Allatostatin, AT: Allatotropin, CAPA: Capability, CCAP: Crustacean cardioactive peptide, CHH: Crustacean hyperglycemic hormone, DH (Cal-like): Calcitonin-like diuretic hormone, DH (CRF-like): Corticotropin-releasing factor-like diuretic hormone, EH: Eclosion hormone, ETH: Ecdysis-triggering hormone, GBP: Growth-blocking peptide, GPA2: Glycoprotein hormone alpha2, GPB5: Glycoprotein hormone beta5, NPF: neuropeptide F, NPLP1: Neuropeptide like precursor 1, PBAN: Pheromone biosynthesis activating neuropeptide, PDH: Pigment dispersing hormone, PTTH: Prothoracicotropic hormone, sNPF: Short neuropeptide F. Database means silk databank (http://silkworm.swu. edu.cn/silk db/) and NCBI. The numbers with “gi “are from NCBI, and the rest from silk databank.
