# Supplementary figures and images for: Microarray-based gene expression profiles of silkworm brains
Source: BMC Neurosci. 2011 Jan 19;12:8. doi: 10.1186/1471-2202-12-8 (PMC3032748; doi:10.1186/1471-2202-12-8)

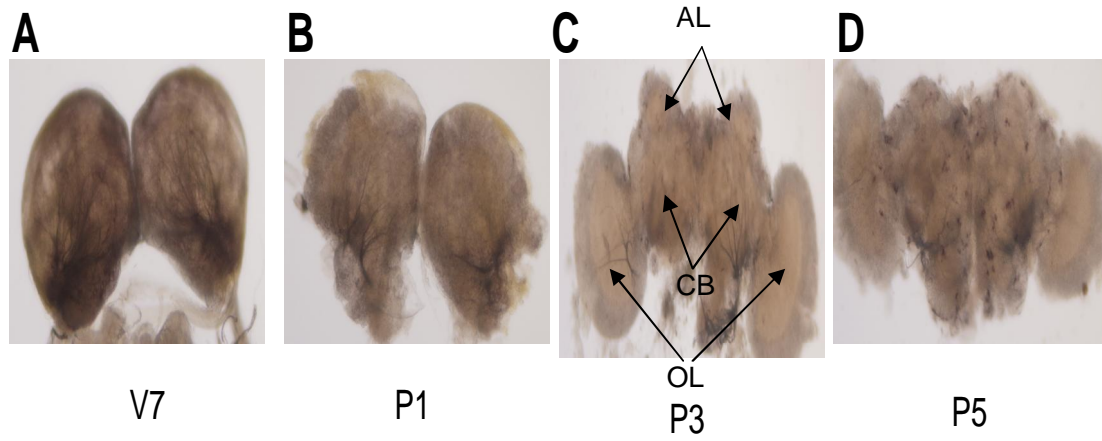

Supplement: Additional file 5 — The morphologic images of silkworm brains at V7, P1, P3 and P5 stages. The file contains the morphologic images of silkworm brains at V7, P1, P3 and P5 stages. The arrow heads indicate the optical lobe (OL), antennal lobe (AL), and central brain (CB), respectively. [file 1471-2202-12-8-S5.PDF]
